# Geophagia in pregnancy and its association with nutritional status - A prospective cohort study in rural north-eastern Tanzania

**DOI:** 10.1186/s12966-025-01721-y

**Published:** 2025-03-04

**Authors:** Erica E. Eberl, Daniel T. R. Minja, Lise E. Lundtoft, Sofie L. Moeller, John P. A. Lusingu, Ib C. Bygbjerg, Inge Tetens, Christentze Schmiegelow, Marta Guasch-Ferré, Dirk L. Christensen, Ruth J.F. Loos, Line Hjort

**Affiliations:** 1 Novo Nordisk Foundation Center for Basic Metabolic Research, Faculty of Health and Medical Sciences, University of Copenhagen, Copenhagen, Denmark; 2 Department of Epidemiology Research, Statens Serum Institut, Copenhagen, Denmark; 3 National Institute for Medical Research, Tanga Centre, Tanga, Tanzania; 4 Global Health Section, Department of Public Health, University of Copenhagen, Copenhagen, Denmark; 5 Department of Nutrition, Exercise and Sports, University of Copenhagen, Copenhagen, Denmark; 6 Centre for translational Medicine and Parasitology, Department of Immunology and Microbiology, Department of Infectious Diseases, University of Copenhagen, Copenhagen University Hospital, Copenhagen, Denmark; 7 Department of Gynecology and Obstetrics, Copenhagen University Hospital– North Zealand, Hillerød, Denmark; 8 Department of Nutrition, Harvard TH Chan School of Public Health, Boston, MA USA; 9 Epidemiology Section, Department of Public Health, University of Copenhagen, Copenhagen, Denmark; 10 Department of Environmental Medicine and Public Health, Icahn School of Medicine at Mount Sinai, New York, NY USA; 11 The Charles Bronfman Institute for Personalized Medicine, Icahn School of Medicine at Mount Sinai, New York, NY USA; 12 Center for Pregnant Women with Diabetes, Department of Obstetrics, Copenhagen University Hospital, Copenhagen, Denmark

**Keywords:** Geophagia, Pica, Pregnancy, Iron status, Folate status, Vitamin B12 status, Mid-upper arm circumference, Anemia, Tanzania

## Abstract

**Background:**

Geophagia or soil-eating behavior is common among pregnant women in sub-Saharan Africa, however its relationship with nutritional status demands further investigation. Using a prospective pregnancy cohort from north-eastern Tanzania, we examined the characteristics of geophagia and its association with nutritional status parameters (mid-upper arm circumference (MUAC), vitamin B12, folate, ferritin, and hemoglobin) before conception and throughout the gestational period.

**Methods:**

Pregnant women (*n* = 530) were interviewed in each trimester regarding their soil-eating habits. Serum concentrations of vitamin B12, folate, ferritin, and hemoglobin, and MUAC were measured before conception and in each trimester. Cross-sectional comparisons between women who ate and did not eat soil were analyzed using Welch’s t-test for continuous variables and χ2-test for categorical variables. The association between changes in nutritional status parameters and the initiation of geophagia was investigated using multivariable logistic regression.

**Results:**

The prevalence of geophagia in this cohort was 27% (*n* = 143) with most women initiating geophagia in the third trimester. Pregnant women that ate soil had significantly lower ferritin (*p* = 0.001) prior to conception and at concentrations diagnostic of iron deficiency (*p* = 0.022) compared to women who did not eat soil. Geophagia was associated with lower ferritin (*p* ≤ 0.001) and lower hemoglobin (*p* < 0.05) in each trimester and lower folate in the third trimester (*p* = 0.007). A smaller decline in hemoglobin and folate across the gestational period was associated with reduced odds of initiating geophagia in the third trimester (hemoglobin: OR 0.71, *p* = 0.008; folate: OR 0.97, *p* = 0.008). There was no significant association between a change in MUAC, serum B12 or ferritin and the initiation of geophagia during pregnancy.

**Conclusions:**

Prenatal geophagia is closely related to iron and folate status. A greater decrease in hemoglobin and folate is associated with the initiation of geophagia during pregnancy. These findings are particularly relevant to low- and middle-income settings where geophagia is practiced and the prevalence of anemia in pregnancy is high.

**Supplementary Information:**

The online version contains supplementary material available at 10.1186/s12966-025-01721-y.

## Background

Geophagia is the deliberate consumption of soil, clay or other earth materials and has existed for centuries worldwide [1]. Today, it is most commonly observed among children and pregnant women as a form of pica [2], an eating disorder characterized by the persistent eating of non-nutritive substances [3]. The prevalence of geophagia in pregnancy (GiP) varies between and within countries but is estimated globally to be 36% with up to 73% in some sub-Saharan African populations [4]. Underreporting is likely as pregnant women may fear judgement for admitting an aberrant eating behaviour and investigators may lack the knowledge and skills to sensitively inquire about it in the context of differing perceptions, beliefs, and cultural norms [5]. GiP is considered a traditional and normal practice in several African countries [6, 7], where soil is often carefully selected and sold for human consumption [2, 4, 8]. In Tanzania, pregnant women have reported eating over 50 g of soil per day, including hardened soil sticks sold at local markets, soil from walls of houses, agricultural fields, termite mounds, and ground soil [9–12]. Cultural beliefs, nutrient supplementation and medicinal use are the traditional incentives for geophagia in Africa [7, 8, 13]. Pregnant women’s personal motives include satisfying cravings, a liking to the taste, texture, or smell of soil, mood improvement, and relief from hypersalivation and gastrointestinal upsets [4, 8, 11, 14]. Despite its sociocultural acceptance, GiP is discouraged by healthcare professionals due to the negative risks far outweighing the possible health benefits [7, 8, 15]. Soil is a source of pathogenic microbes, parasites, environmental pollutants, and heavy metals [16–19], all of which can be deleterious to both the mother and offspring [20–22]. Cases of excessive tooth abrasion and enamel damage [23, 24], as well as colonic obstruction and perforation [25–27], have also been attributed to geophagia.

It is well known that geophagia is strongly associated with anemia [28] and is negatively correlated with hemoglobin, ferritin, and hematocrit levels among pregnant women in Kenya and Tanzania [29, 30]. Iron requirements increase during pregnancy, and a failure to maintain sufficient levels is associated with adverse gestational outcomes, including placental abruption, preterm birth, fetal malformation and growth restriction [31, 32]. However, whether GiP is the result or cause of poor iron status is an ongoing debate, largely due to the lack of randomized controlled trials and longitudinal research [2, 6, 33]. Its association with anemia also suggests a possible relationship with folate or vitamin B12 deficiency [34], yet no studies have prospectively examined this hypothesis despite these nutrients playing a pivotal role in maternal and fetal development [35, 36]. Furthermore, no studies have examined the association between GiP and markers of malnutrition in pregnancy, such as mid-upper arm circumference (MUAC) [37, 38], and no studies have investigated the relationship between GiP and nutritional status before pregnancy or in early first trimester. These areas are important in understanding the significance and prevention of this eating behavior.

Using data from a prospective pregnancy cohort in rural northeastern Tanzania, this study examined the characteristics of GiP and its association with nutritional status before conception and in each trimester. To further elucidate the relationship, a secondary aim was to determine whether changes in MUAC, vitamin B12, folate, ferritin, or hemoglobin concentrations across pregnancy are associated with the initiation of GiP.

## Methods

### Study design and population

**Fig. 1:**
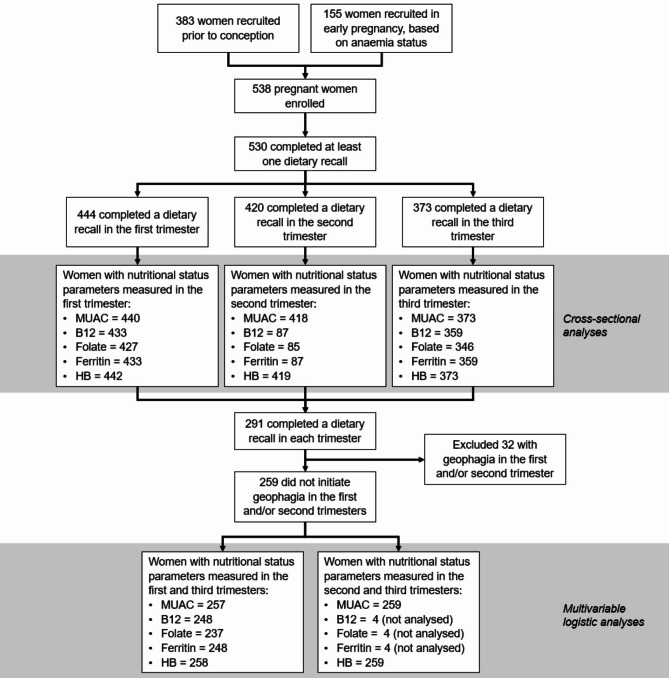
Participant flowchart and inclusion criteria for study analyses. The association between nutritional status parameters and geophagia in each trimester was analyzed cross-sectionally using Welch’s t-test and chi-squared test. The association between the initiation of geophagia in the third trimester and a change in nutritional status from either the first or second trimester was analyzed using multivariable logistic regression

We used data from the FOETALforNCD Study (FOetal exposure and Epidemiological Transition: the role of Anemia in early Life for Non-Communicable Diseases in later life), the details of which have been published elsewhere [39]. From July 2014 to December 2016, 538 pregnant women from Korogwe and Handeni districts, Tanga region, northeastern Tanzania, were enrolled in the study. Women were either part of a preconception cohort that were recruited before pregnancy or a pregnancy cohort that were selectively recruited in early pregnancy (≤ 14 gestational weeks) based on anemia status. This was done to ensure the pregnancy cohort consisted of equal numbers of women with and without anemia (defined as hemoglobin < 11.0 g/dL or ≥ 11.0 g/dL respectively) to meet the FOETALforNCD cohort study’s objectives. The FOETALforNCD inclusion and exclusion criteria have been described and justified previously [39]. In short, the inclusion criteria for women recruited at pre-conception were: age between 18 and 40 years, negative urine pregnancy test, living in an accessible area for the study, and should conception occur, willing to attend antenatal care and give birth at Korogwe District Hospital (KDH). Exclusion criteria included unsuccessful attempts to conceive for more than two consecutive years, usage of modern contraceptive methods except condoms, and having a child less than 9 months old. The women were invited to report for pregnancy testing every third month or if they suspected they were pregnant. Women were invited to participate in the pregnancy study if conception occurred between July 2014 and March 2016. Pregnant women that had not been recruited prior to conception were screened for anemia status and recruited between April 2015 to March 2016. The pregnancy cohort therefore consisted of 383 women recruited prior to conception and 155 women recruited in early pregnancy (78 with anemia and 77 without) (Fig. 1). Women were followed throughout pregnancy with scheduled antenatal care (ANC) visits at gestational weeks 11–14, 20–22, 26–28, 32–34 and 37–39. Follow-up of pregnant women was completed in December 2016 when the last woman in the cohort gave birth.

### Point-of-care

In accordance with the ANC service in Tanzania, all pregnant women were offered to take 200 mg ferrous sulfate (equivalent to 43 mg ferrous iron) and 400 µg folic acid daily (Ferrolic–LF, Laboratory and Allied LTD, Mombasa, Kenya). If diagnosed with anemia at any ANC visit, women were either advised to increase supplementation or take Hemovit multivitamin syrup (Shelys Pharmaceuticals, Dar es Salaam, Tanzania) depending on anemia severity [39]. The minimum recommended dose of Hemovit amounted to 800 mg ferrous sulfate, 2 mg vitamin B6, 200 µg vitamin B12, 6 mg folic acid, and 9.32 mg zinc sulphate per day. Intermittent preventive treatment in pregnancy for malaria was provided from gestational week 16, and empirical treatment of helminth infestations at gestational week 20. Malaria was treated with quinine, artemether-lumefantrine, or artesunate injections, according to national guidelines and depending on the severity of illness and gestational age [39].

### Data collection

The primary site of data collection was the maternity ward and reproductive and child health clinic at KDH. Clinical investigations and sample collection also took place via outreach dispensaries and mobile clinics across the Korogwe and Handeni districts. At the first ANC visit (enrolment), trained staff, fluent in Kiswahili, interviewed women and collected information regarding sociodemographic characteristics and medical history, including obstetrics. Socioeconomic status (SES) was determined using principal components analysis, based on the woman’s educational level, occupation, and economic characteristics [40]. The respective SES scores were categorized in tertiles as low, medium, and high, with low SES scores given for no or partial primary education, housewife or farmer as occupation, pond or river as primary water source, rental housing, thatch roofing, and having no toilet [40]. HIV infection was tested and recorded at enrolment. Anthropometric data, including MUAC, as well as information about medication and supplement use, malaria, gestational diseases and complications were obtained at enrolment and each subsequent ANC visit.

Venous blood was collected during ANC visits in EDTA and serum tubes and kept at 2–8 °C until processed at KDH within 2 h of collection. Hemoglobin was measured at all ANC visits using a Sysmex KX-21 N hematological analyzer (Sysmex Corporation, Kobe, Japan). Serum ferritin, folate, and vitamin B12 concentrations were measured at enrolment and 32–34 gestational weeks. Ferritin was measured using Vitros DT60II chemistry system (Diamond Diagnostics, Massachusetts, USA), and folate and vitamin B12 were measured using Vista 1500 chemical analyzer (Siemens Healthcare, Erlangen, Germany).

Information about GiP was collected as part of a structured dietary recall, conducted by trained personnel in Kiswahili at enrolment and once per subsequent trimester. During the dietary recall, women were asked if they had eaten soil at any time point since the last dietary assessment, or since becoming pregnant if it was the first dietary recall, and to provide details, including the frequency of consumption (number of eating occasions per day, week, or month), portion size, and type of soil consumed (ground soil, soil-sticks, or hardened clay). Portion sizes were quantified with the aid of measuring utensils and pictures developed by the research team (see Supplementary Fig. 1, Additional File 1). Each dietary recall was checked for completeness and any missing data was followed up at the next ANC visit.

### Statistical analysis

For all analyses, the definition of first trimester was < 14 gestational weeks, second trimester ≥ 14 and < 28 gestational weeks, and third trimester ≥ 28 gestational weeks. All tests were 2-tailed and p-values < 0.05 were considered significant. We first investigated associations of GiP with baseline and demographic characteristics. Welch’s t-test was used to compare the means of continuous variables, and Pearson’s chi-squared (χ2) test was used to compare the distributions of categorical variables between women with and without GiP.

### GiP characteristics

Characteristics of geophagia were summarized cross-sectionally at each trimester where GiP was reported. Soil type was reported in counts and percentages, and the portion sizes, frequencies, and daily intakes were summarized by means and 95% CI. In the case a woman reported geophagia in more than one diet recall within the same trimester, only the soil type reported in the first recall for that trimester was considered and the frequency and amount consumed were calculated as averages from the repeated reports. The temporality of GiP and GiP initiation was examined among women who completed a dietary recall in all three trimesters and reported geophagia in at least one. An UpSet plot was used to visualize the number of women that reported GiP in one and multiple trimesters.

### Pre-pregnancy nutritional status

A subsample of the pregnancy cohort had nutritional status parameters measured before conception, including anthropometric measurements (MUAC, BMI) and nutrient biomarkers (vitamin B12, folate, ferritin, hemoglobin) (*n* = 328). Differences in nutritional status parameters and proportions of those deficient at pre-pregnancy were compared between women with and without GiP at any time during pregnancy. Vitamin B12 deficiency was defined as serum B12 < 150pmol/mL and folate deficiency as serum folate < 10nmol/L [41]. Iron deficiency was defined as serum ferritin concentration < 15ug/L [42] after using a correction factor of 0.67 if CRP > 5 mg/L [43]. Anemia was defined as hemoglobin < 12g/dL for non-pregnant women [44]. Ferritin was log-transformed prior to statistical testing due to skewed distribution. Data were summarized by means and 95% confidence intervals for nutritional status parameters and counts and percentages for nutritional deficiencies.

### Nutritional status in each trimester

Cross-sectional associations between GiP and nutritional status parameters, excluding BMI, were assessed in each trimester using the same analytical approach. In the event a woman completed more than one dietary recall within the same trimester, the first recall or the first recall that reported geophagia was considered (see Supplementary Fig. 2, Additional File 1). In the event a women had a nutritional status parameter measured more than once within the same trimester, only the measurement taken at the time of the dietary recall was included in the analyses, excluding measurements taken at delivery. If no measurements were taken at the time of dietary recall, then the measurement taken at the previous visit within the same trimester, or following visit if no previous visits occurred, was included (see Supplementary Fig. 3, Additional File 1). Anemia was defined as < 11g/dL in the first and third trimesters, and < 10.5g/dL in the second trimester [44]. The means and distributions of nutritional status parameters in each trimester were visualized using violin plots.

### Changes and initiation of GiP

**Fig. 2:**
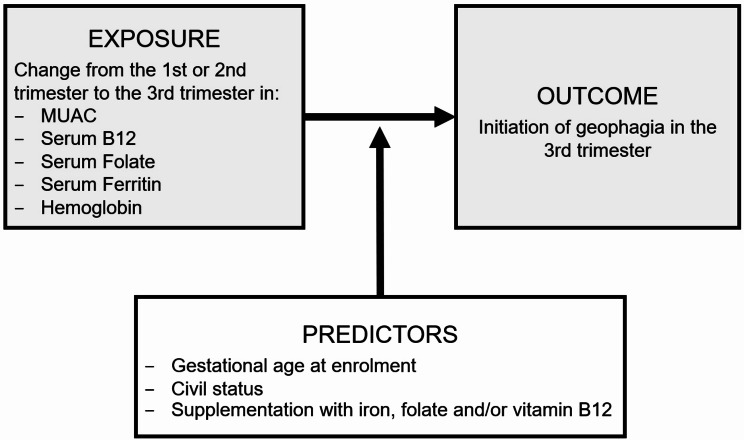
Diagram of logistic regression analysis model. Predictors were selected using stepwise backward selection from the following list of co-variates: age, parity, maternal occupation, history of miscarriage, gestational age at third trimester visit, enrolment year, supplementation with iron, folate and/or vitamin B12 at enrolment, malaria at any time during pregnancy, use of antihelminth medication at any time during pregnancy, number of scheduled ANC visits during the study, preeclampsia. The change in each nutritional status parameter from first or from second trimester and its association with the initiation of geophagia in the third trimester was tested in separate logistic regression models, adjusting for gestational age at enrolment, civil status and supplementation with iron, folate, and/or B12 at any time during pregnancy

Logistic regression was used to investigate the association between the initiation of GiP and changes in nutritional status parameters across the gestational period. Since too few women reported GiP in the first and second trimester, only the initiation of GiP in third trimester was considered. Women with GiP in the first and/or second trimesters were excluded from the analyses such that any geophagia reported in the third trimester could be considered a new behavior initiated during pregnancy. Differences in MUAC and serum concentrations of vitamin B12, folate, ferritin, and hemoglobin, from the first to the third trimester, were tested in separate multivariable logistic regression models with geophagia in the third trimester as the dependent variable. Covariates were selected from univariate analyses (*p* < 0.25) [45] with GiP in any trimester as the dependent variable (*n* = 530) and GiP in the third trimester as the dependent variable (*n* = 373). Stepwise backward selection was used to determine the following predictors to adjust for: gestational age at first ANC visit, civil status, and supplementation with Hemovit multivitamin syrup or vitamin B12 at any time during pregnancy(Fig. 2). The same multivariable logistic regression model was used to analyze the association between the initiation of geophagia in the third trimester and changes in MUAC and hemoglobin from the first to the second trimester and the second to the third trimester. Starting concentrations of nutritional status parameters could influence the association between changes of such and the initiation of GiP. To test for this, we used Welch’s t-test to compare the mean concentrations and χ2-test to compare the proportion of deficient women in the first and second trimester between those that initiated and did not initiate GiP in the third trimester. All statistical analyses and graphical illustrations were conducted using RStudio software (version 2024.4.2.764).

## Results

### Demographics and baseline characteristics associated with GiP

Of the 538 pregnant women enrolled, 530 women completed at least one dietary recall during pregnancy with 291 (55%) completing a dietary recall in all three trimesters (see Supplementary Fig. 4, Additional File 1). The proportion of women with GiP at any time during pregnancy was 27% (143/530). The average gestational age at enrolment, during the first ANC visit, was earlier for women who never ate soil (9.5 weeks, 95%CI = [9.1;9.9]) compared to those who ate soil at any time during pregnancy (11.1 weeks, 95%CI = [10.3; 11.9], *p* = 6.51e-4) (Table 1). A greater proportion of women with GiP were taking supplements containing iron and folic acid at enrolment (6.3%) compared to women without GiP (1.6%, *p* = 0.009). There was no significant difference between women with and without GiP regarding age, season at enrolment, parity, history of miscarriage, history of stillbirth, civil status, ethnicity, reigion, education level, occupation, SES, and chronic disease. At enrolment, there was also no significant difference regarding weight, BMI, MUAC, alcohol intake, medication use, anemia and recent history of anemia, malaria infection, and HIV status. No women in the cohort smoked.

**Table 1:** Demographics and baseline characteristics of women with and without geophagia at any time during pregnancy

Characteristic^1^	Overall^2^ *n* = 530	No GiP^2^ *n* = 387	GiP^2^ *n* = 143	*p*-value^3^
**Age** (years)	27.8 [27.2, 28.3]	28.0 [27.3, 28.6]	27.3 [26.2, 28.4]	0.272
**Gestational age at enrolment** (weeks)	10.0 [9.6, 10.3]	9.5 [9.1, 9.9]	11.1 [10.3, 11.9]	**6.51e-4**
**Season at enrolment**				0.662
Dry (Dec-Mar)	248 (46.8%)	179 (46.3%)	69 (48.3%)	
Long rains (Apr-May)	52 (9.8%)	36 (9.3%)	16 (11.2%)	
Harvest (Jun-Sep)	146 (27.5%)	112 (28.9%)	34 (23.8%)	
Short rains (Oct-Nov)	84 (15.8%)	60 (15.5%)	24 (16.8%)	
**Weight** (kg)	57.6 [56.6, 58.7]	57.8 [56.6, 59.0]	57.2 [55.1, 59.4]	0.661
**BMI** (kg/m^2^)	23.8 [23.4, 24.2]	23.8 [23.4, 24.3]	23.8 [23.0, 24.5]	0.901
**MUAC** (cm)	28.3 [28.0, 28.7]	28.4 [28.0, 28.8]	28.2 [27.5, 28.8]	0.509
**Alcohol intake**				0.863
Yes	12 (2.3%)	8 (2.1%)	4 (2.8%)	
No	518 (97.7%)	379 (97.9%)	139 (97.2%)	
**Parity**				0.111
Nulliparous	67 (12.6%)	45 (11.6%)	22 (15.4%)	
Primiparous	114 (21.5%)	77 (19.9%)	37 (25.9%)	
Multiparous	349 (65.8%)	265 (68.5%)	84 (58.7%)	
**History of miscarriage**				0.304
Yes	109 (20.6%)	75 (19.4%)	34 (23.9%)	
No	420 (79.4%)	312 (80.6%)	108 (76.1%)	
**History of stillbirth**				0.507
Yes	34 (6.5%)	27 (7.0%)	7 (4.9%)	
No	493 (93.5%)	358 (93.0%)	135 (95.1%)	
**Civil status**				0.348
Married	453 (86.0%)	334 (87.0%)	119 (83.2%)	
Partner, co-habiting	23 (4.4%)	13 (3.4%)	10 (7.0%)	
Partner, non-cohabiting	47 (8.9%)	34 (8.9%)	13 (9.1%)	
Single, divorced, widowed	4 (0.8%)	3 (0.8%)	1 (0.7%)	
**Ethnicity**				0.788
Sambaa	194 (36.6%)	144 (37.2%)	50 (35.0%)	
Zigua	172 (32.5%)	126 (32.6%)	46 (32.2%)	
Pare	37 (7.0%)	28 (7.2%)	9 (6.3%)	
Bondei	15 (2.8%)	9 (2.3%)	6 (4.2%)	
Other	112 (21.1%)	80 (20.7%)	32 (22.4%)	
**Religion**				0.447
Islamic	396 (75.1%)	284 (73.8%)	112 (78.9%)	
Catholic	30 (5.7%)	24 (6.2%)	6 (4.2%)	
Lutheran	26 (4.9%)	22 (5.7%)	4 (2.8%)	
Anglican	50 (9.5%)	35 (9.1%)	15 (10.6%)	
Other	25 (4.7%)	20 (5.2%)	5 (3.5%)	
**Education**				0.619
None	49 (9.2%)	37 (9.6%)	12 (8.4%)	
Partial primary	72 (13.6%)	48 (12.4%)	24 (16.8%)	
Complete primary	353 (66.6%)	261 (67.4%)	92 (64.3%)	
Secondary or more	56 (10.6%)	41 (10.6%)	15 (10.5%)	
**Occupation**				0.357
Professional	9 (1.7%)	5 (1.3%)	4 (2.8%)	
Business	59 (11.2%)	46 (11.9%)	13 (9.1%)	
Service	17 (3.2%)	12 (3.1%)	5 (3.5%)	
Farmer	357 (67.6%)	263 (68.3%)	94 (65.7%)	
Housework	85 (16.1%)	59 (15.3%)	26 (18.2%)	
Other	1 (0.2%)	0 (0.0%)	1 (0.7%)	
**Socioeconomic status** ^*4*^				0.380
Low	184 (35.2%)	129 (33.6%)	55 (39.6%)	
Middle	168 (32.1%)	124 (32.3%)	44 (31.7%)	
High	171 (32.7%)	131 (34.1%)	40 (28.8%)	
**Malaria infection**				0.652
Positive	71 (13.5%)	54 (14.1%)	17 (12.1%)	
Negative	454 (86.5%)	330 (85.9%)	124 (87.9%)	
**HIV status**				0.925
Positive	17 (3.4%)	13 (3.6%)	4 (2.9%)	
Negative	478 (96.6%)	346 (96.4%)	132 (97.1%)	
**Chronic disease** ^*5*^				0.520
Yes	76 (14.6%)	53 (13.9%)	23 (16.7%)	
No	443 (85.4%)	328 (86.1%)	115 (83.3%)	
**Anemia**				0.134
Yes	119 (22.5%)	80 (20.7%)	39 (27.3%)	
No	411 (77.5%)	307 (79.3%)	104 (72.7%)	
**History of anemia** ^*6*^				1.000
Yes	6 (1.1%)	4 (1.0%)	2 (1.4%)	
No	522 (98.9%)	381 (99.0%)	141 (98.6%)	
**Medication use** ^*7*^				1.000
Yes	37 (7.0%)	27 (7.0%)	10 (7.0%)	
No	492 (93.0%)	360 (93.0%)	132 (93.0%)	
**Supplement use** ^*8*^				**0.009**
Yes	15 (2.8%)	6 (1.6%)	9 (6.3%)	
No	515 (97.2%)	381 (98.4%)	134 (93.7%)	

Abbreviations: GiP, geophagia in pregnancy; MUAC, mid-upper arm circumference

^*1*^All characteristics were collected at study enrolment during the first antenatal care visit

^*2*^Mean [95% CI] or frequency (%)

^*3*^Welch two sample t-test for continuous variables; Pearson’s χ2-test for categorical variables. P-values < 0.05 are shown in bold

^*4*^Socio-economic status was determined using principal components analysis based on maternal education, occupation, source of domestic water, type of house ownership, roofing materials and type of toilet facility

^*5*^Chronic diseases self-reported at enrolment were gastric ulcers, hypertension, diabetes, asthma, chronic kidney disease, epilepsy, cardiomegaly, hypotension, and lymphatic filariasiz

^*6*^Self-reported anemia within 6 months prior to the enrolment

^*7*^Medications included antibiotics, antimalarials, antiretrovirals, antihypertensives, anti-asthmatics, painkillers (paracetamol, aspirin, diclofenac), omeprazole, phenobarbital

^*8*^Supplements taken within 2 months prior to enrolment were a combined iron and folic acid supplement or multivitamin syrup (Hemovit)

### Characteristics of GiP

Overall, women with GiP ate 37.7g of soil 1.6 times per day (Table 2). The average daily intake was 70.7g (95% CI = 50.0; 91.4, Table 2) and ranged from 1.3g to 1245g per day (data not shown). When examined cross-sectionally, the average daily intake of soil increased with each trimester, with the greatest amount of soil consumed in the third trimester (Table 2). The prevalence of GiP also increased with gestational age with 5% (20/444) of women in the first trimester eating soil, 8% (34/420) in the second trimester, and 33% (122/373) in the third trimester (Table 2; see Supplementary Fig. 5, Additional File 1). Soil-sticks were the preferred soil type in the first trimester while clay was the preferred soil type in the second and third trimesters (Table 2).

**Table 2:** Soil intake characteristics among women with geophagia in first, second, and/or third trimester

	**Trimester**			**Overall** *n* = 143^*2*^
**Characteristic**	**First**	**Second**	**Third**	
	*n* = 20^*1*^	*n* = 34^*1*^	*n* = 122^*1*^	
**Soil type**				
Clay	6 (30.0%)	23 (67.6%)	71 (58.2%)	100 (69.9%)
Soil-stick	11 (55.0%)	8 (23.5%)	34 (27.9%)	53 (37.1%)
Ground soil	1 (5.0%)	1 (2.9%)	8 (6.6%)	10 (7.0%)
**Portion size** (g)	22.4 [16.4, 28.4]	44.0 [29.2, 58.8]	40.8 [32.0, 49.6]	37.7 [32.6, 42.9]
**Daily frequency** (portions/day) ^*3*^	1.3 [0.9, 1.6]	1.2 [0.9, 1.5]	1.7 [1.5, 1.9]	1.6 [1.4, 1.8]
**Daily intake** (g/day)	36.3 [16.3, 56.4]	54.5 [35.1, 73.8]	88.3 [44.0, 132.6]	70.7 [50.0, 91.4]

1 Mean [95%CI] or frequency (%) among women who reported eating soil in the first, second or third trimester-

^*2*^Mean [95%CI] or frequency (%) among women who reported eating soil at any time during pregnancy. Percentages sum to greater than 100 due to some women eating more than one type of soil across the gestational period. Average intake per individual was calculated before the average among all individuals

^*3*^Daily frequency as reported or calculated from weekly frequency or monthly frequency

**Fig. 3:**
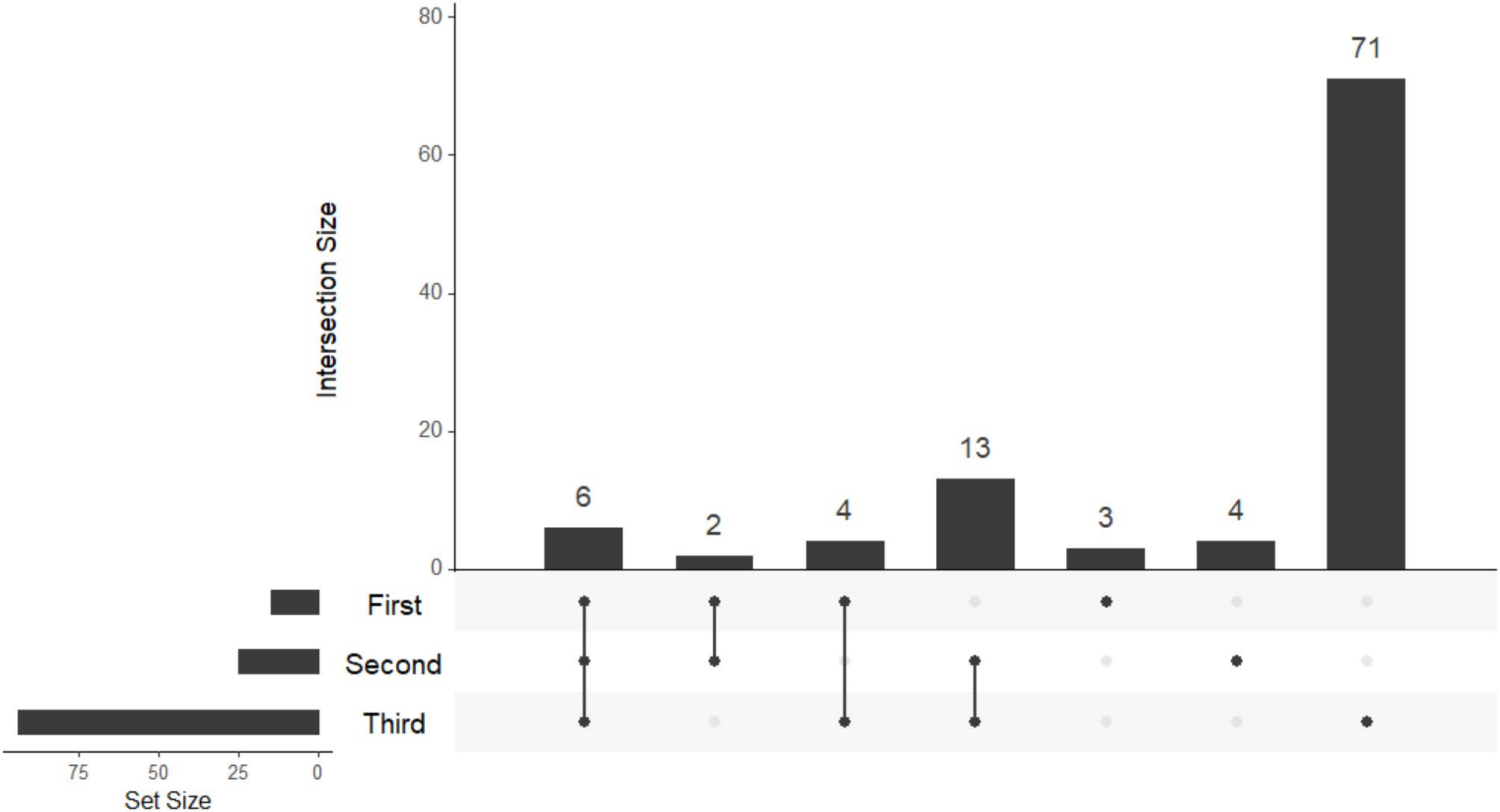
Trimesters of geophagia occurrence. UpSet plot showing the number of women that reported geophagia in the first, second and/or third trimester among those who completed a dietary recall in all three trimesters and reported geophagia in at least one (*n* = 103). The set size corresponds to the total number of women with geophagia in a certain trimester. The intersection size corresponds to the number of women with geophagia in one or more trimester(s), with the dots under each column specifying the trimester(s)

We further examined the temporality of GiP among women who completed a dietary recall in all three trimesters and reported geophagia in at least one of these recalls (*n* = 103). Of these women, 15% ate soil in the first trimester, 24% in the second trimester, and 91% in the third trimester (Fig. 3). Most women did not eat any kind of soil in more than one trimester, with 69% initiating geophagia in the third trimester.

### Pre-pregnancy nutrient status and GiP

To investigate an association between GiP and nutritional status prior to conception, we compared anthropometric measurements and serum concentrations of nutrient biomarkers taken before pregnancy between women who eventually ate or did not eat soil during pregnancy. On average, women with GiP in any trimester had significantly lower ferritin (*p* = 0.001) and had a greater proportion with iron deficiency at pre-pregnancy (*p* = 0.022) compared to women without GiP (Table 3). There was no significant difference in pre-pregnancy BMI, MUAC, vitamin B12, folate, or hemoglobin between women with and without GiP.

**Table 3:** Pre-pregnancy nutritional status parameters among women with or without geophagia at any time during pregnancy

Pre-pregnancy parameter^1^		Geophagia in pregnancy			*p*-value^3^
	**n**	No^2^	**n**	Yes^2^	
**BMI** (kg/m^2^)	182	24.3 [23.6, 25.0]	73	23.5 [22.3, 24.6]	0.209
**MUAC** (cm)	183	29.1 [28.5, 29.7]	73	28.3 [27.3, 29.4]	0.182
**Vitamin B12** (pmol/L)	225	547.9 [515.5, 580.2]	83	535.5 [481.9, 589.1]	0.696
Deficient (< 150pmol/L)		1 (0.4%)		0 (0%)	1.000
**Folate** (nmol/L)	216	34.4 [32.1, 36.6]	80	31.2 [28.4, 34.0]	0.079
Deficient (< 10nmol/L)		4 (1.9%)		2 (2.5%)	1.000
**Ferritin** (µg/L)	225	29.4 [25.7, 33.1]	83	19.5 [16.1, 23.0]	**0.001** ^*4*^
Defcient (< 15µg/L)		72 (32%)		39 (47%)	**0.022**
**Hemoglobin** (g/dL)	186	12.3 [12.1, 12.5]	73	12.1 [11.8, 12.5]	0.381
Anemia (< 12g/dL)		65 (35%)		26 (36%)	1.000

Abbreviations: MUAC, Mid-upper arm circumference

^*2*^Mean [95%CI] or frequency (%)

^*3*^Welch two sample t-test for continuous variables; Pearson’s χ2-test for categorical variables. P-values < 0.05 are shown in bold

^*4*^p-value after log-transformation of corrected ferritin concentrations

^*1*^Nutritional parameters were all measured prior to conception from women that were recruited pre-conceptively and were enrolled in the pregnancy study. A correction factor of 0.67 was applied to ferritin if CRP > 0.5mg/L at the time of measurement. BMI = body mass index

### Nutritional status and GiP in each trimester

**Fig. 4:**
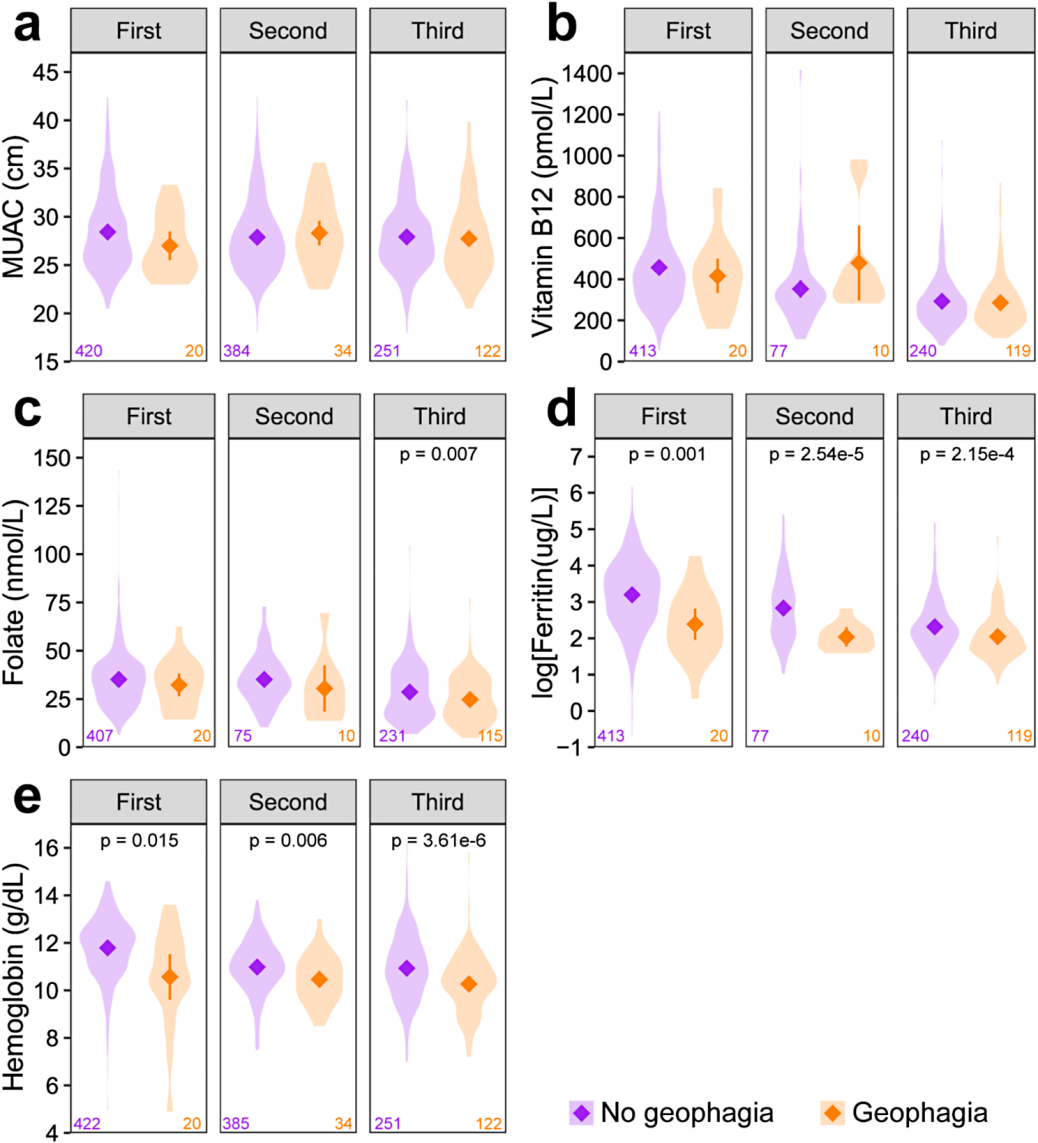
Measurements of nutritional status parameters among women with and without geophagia in each trimester. Data points are means with 95% CIs. Cross-sectional comparisons of nutritional status parameters were analyzed using Welch’s t-test, p-values < 0.05 are shown. Ferritin was corrected for inflammation using a correction factor of 0.67 if CRP > 0.5 mg/L at the time of measurement and log-transformed for statistical analysis. Distributions of the individual datapoints for each group are shaded with sample sizes shown in the bottom corners of each plot

**Fig. 5:**
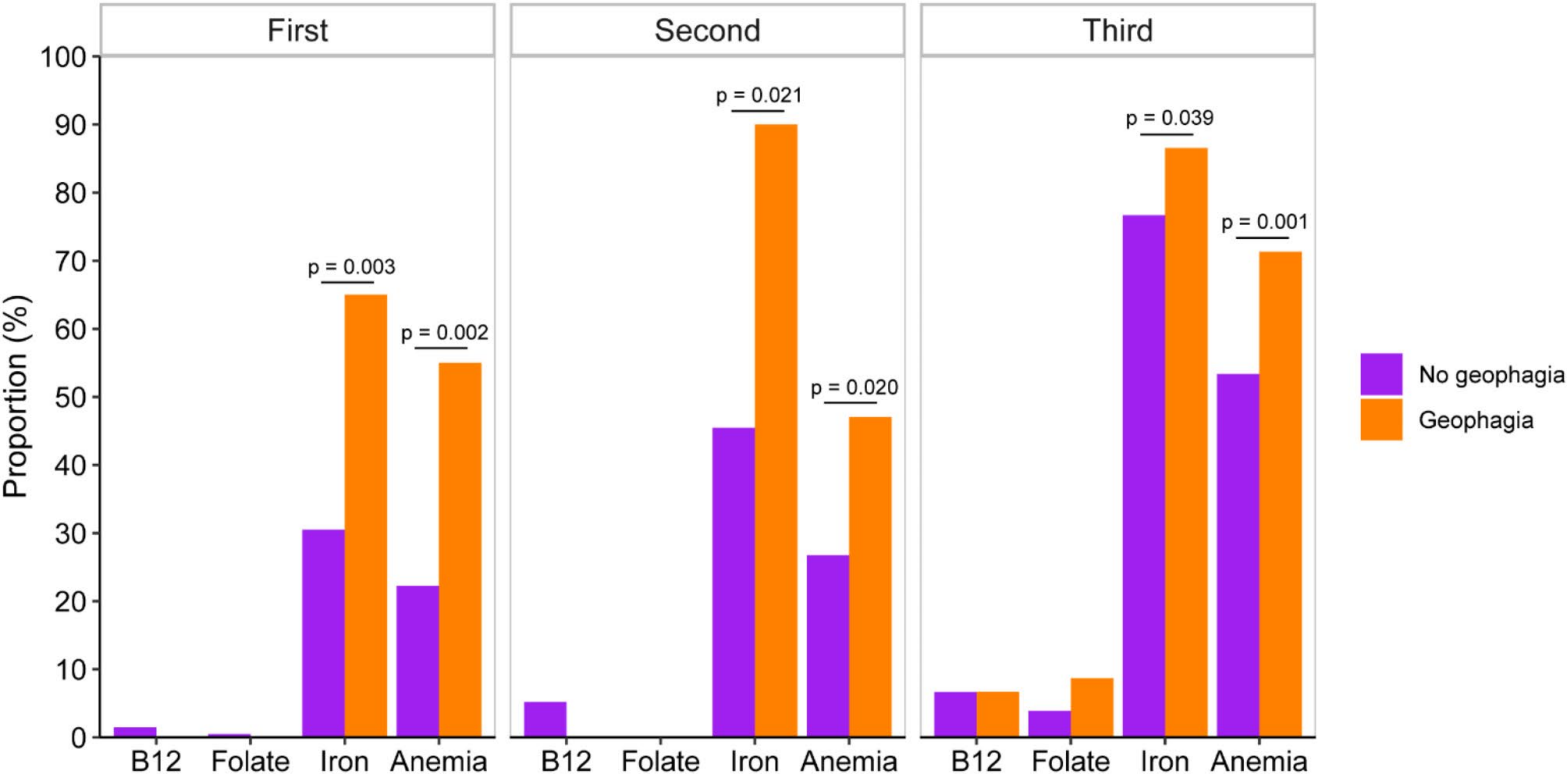
Proportions of nutritional deficiencies among women with and without geophagia in each trimester. Differences in the distribution of proportions of deficiencies between women who did and did not eat soil in each trimester were analyzed using Pearson’s χ2-test. P-values < 0.05 are shown. Vitamin B12 deficiency was defined as serum B12 < 150pmol/mL, folate deficiency as serum folate < 10nmol/L, iron deficiency as serum ferritin < 15ug/L after using a correction factor of 0.67 if CRP > 5mg/L at the time of measurement, and anemia as < 11g/dL in the first and third trimesters and < 10.5g/dL in the second trimester

There was no significant difference in MUAC or vitamin B12 between women with or without GiP at any time-point during pregnancy (Fig. 4a-b). In the third trimester, women who ate soil had significantly lower folate than women who did not eat soil (*p* = 0.007, Fig. 4c) although there was no difference in the proportion of those that were folate deficient (Fig. 5). There was no significant difference in folate between women with and without GiP in the first or second trimesters. In each trimester, women who ate soil had lower ferritin compared to women who did not eat soil in the same trimester and the difference was significant after log-transformation (first trimester: *p* = 0.001, second trimester: *p* = 2.54e-5, third trimester: *p* = 2.15e-4, Fig. 4d). A greater proportion of women with GiP were iron deficient in each trimester compared to women without GiP (first trimester: *p* = 0.003, second trimester: *p* = 0.021, third trimester: *p* = 0.039, Fig. 4) Similarly women with GiP had significantly lower hemoglobin compared to women without GiP (first trimester: *p* = 0.015, second trimester: *p* = 0.006, third trimester: *p* = 3.61e-6, Fig. 4e) and had a greater proportion suffering from anemia (first trimester: *p* = 0.002, second trimester: *p* = 0.020, third trimester: *p* = 0.001, Fig. 4).

### Changes in nutrient status and initiation of GiP in third trimester

Changes in folate and hemoglobin from the first to the third trimester were significantly associated with the initiation of GiP (Table 4). Women who did not start eating soil in the third trimester experienced an average decline of 6.37nmol/L in folate and 0.79g/dL in hemoglobin, while women who started eating soil experienced approximately double the decline in folate and hemoglobin. After adjusting for covariates, it was found that reducing the severity of decline of folate by 1nmol/L would reduce the odds of initiating GiP by 3% (*p* = 0.008), while reducing the severity of decline of hemoglobin by 1g/dL would reduce the odds of initiating GiP by 29% (*p* = 0.008). There was no significant difference in nutritional status parameters measured in the first trimester between those that did and did not initiate GiP (see Supplementary Table 1, Additional File 1). Those that initiated GiP had lower hemoglobin in the second trimester compared to those that did not initiate (*p* = 0.012; see Supplementary Table 2, Additional File 1).

**Table 4:** Changes in nutritional status parameters from first trimester and initiation of geophagia in third trimester

Parameter^1^	Geophagia initiated				OR [95%CI]	AOR [95%CI]^3^	AOR *p*-value
	**n**	No^2^	**n**	Yes^2^			
**MUAC (cm)**	188	-0.36 [-0.60;-0.12]	69	-0.62 [-1.01;-0.23]	0.91 [0.78;1.08]	0.92 [0.78;1.09]	0.340
**B12** (pmol/L)	180	-3.23 [-3.64;-2.82]	68	-3.45 [-4.16;-2.73]	0.97 [0.88;1.07]	0.94 [0.85;1.04]	0.235
**Folate** (nmol/L)	173	-6.37 [-8.48;-4.25]	64	-12.55 [-16.75;-8.35]	0.97 [0.95;0.99]	0.97 [0.95;0.99]	**0.008**
**Ferritin** (µg/L)	180	-2.46 [-3.16;-1.76]	68	-2.80 [-3.95;-1.64]	0.99 [0.93;1.04]	0.98 [0.93;1.04]	0.502
**Hemoglobin** (g/dL)	188	-0.79 [-1.00;-0.58]	70	-1.31 [-1.55;-1.07]	0.71 [0.55;0.91]	0.71 [0.55;0.91]	**0.008**

Abbreviations: AOR, adjusted odds ratio; B12, serum vitamin B12; MUAC, mid-upper arm circumference; OR, crude odds ratio

^*1*^Nutritional status parameters measured in the first and third trimesters. Ferritin was corrected for inflammation using a correction factor of 0.67 if CRP > 0.5mg/L at the time of measurement

^*2*^Mean difference [95%CI] from first to third trimester

^*3*^Adjusted for civil status, gestational age (days) at enrollment, and supplementation with vitamin B12 or multivitamin syrup (Hemovit) during pregnancy. P-values < 0.05 are shown in bold

No significant association was found between changes in hemoglobin from second trimester and the initiation of GiP in third trimester (Table 5); however, after adjusting for covariates, a 1g/dL reduction in the severity of decline of hemoglobin from first to second trimester was associated with a 36% decrease in the odds of initiating GiP (*p* = 0.006; see Supplementary Table 3, Additional File 1). The initiation of GiP was not related to changes in MUAC, vitamin B12, or ferritin during pregnancy.

**Table 5:** Changes in MUAC and hemoglobin from second trimester and initiation of geophagia in third trimester

Parameter^1^	No initiation^2^ (*n* = 188)	Initiation^2^ (*n* = 71)	OR [95%CI]	AOR [95%CI]^3^	AOR *p*-value
**MUAC (cm)**	-0.12 [-0.32;0.09]	-0.24 [-0.54;0.06]	0.94 [0.77;1.14]	0.95 [0.78;1.17]	0.647
**Hemoglobin** (g/dL)	-0.08 [-0.25;0.09]	-0.24 [-0.45;-0.04]	0.87 [0.67;1.12]	0.91 [0.70;1.19]	0.499

Abbreviations: AOR, adjusted odds ratio; MUAC, mid-upper arm circumference; OR, crude odds ratio

^*1*^Nutritional status parameters measured in the second and third trimesters

^*2*^Mean difference [95%CI] from second to third trimester

^*3*^AOR= Adjusted for civil status, gestational age (days) at enrollment, supplementation with vitamin B12 or multivitamin syrup (Hemovit) during pregnancy

## Discussion

In this prospective cohort study, we examined GiP and its relation to MUAC, vitamin B12, folate, and iron status in pre-pregnancy and across the gestational period among women in rural northeastern Tanzania. The prevalence of GiP in the study cohort was 27%, which is consistent with findings from other regions in Tanzania [9, 10, 46]. We found GiP to be associated with lower ferritin concentrations and iron deficiency in pre-pregnancy and in every trimester. GiP was also associated with lower hemoglobin and anemia during pregnancy and lower folate in the third trimester. The initiation of GiP was related to a greater decline in hemoglobin and folate in the period preceding GiP initiation. We found no evidence supporting a relationship between GiP and MUAC or vitamin B12 concentrations, before or during pregnancy.

This is the first study to investigate GiP in relation to folate status. Similar studies considering folate have focused on pica more broadly and with a smaller number of cases [47, 48]. In a cohort of African American women in Washington, lower folate during pregnancy was associated with amylophagia (craving for starch) but not other forms of pica [47]. As the researchers observed no cases of geophagia, is it possible that the association we found between folate status and GiP was either non-pica-related or pica-related in this specific African setting where GiP is more culturally acceptable [8, 12, 13]. Consistent with our findings in pre-pregnancy, previous research has found no difference in folate concentrations between non-pregnant women with or without pica [49]. The relationship between folate and geophagia might be specific to gestation, where folate requirements are higher to facilitate DNA replication and foetal development. However more studies are needed to confirm this relationship.

Our results support previous findings that GiP is an important indicator of iron deficiency and anemia during pregnancy [28–30, 50, 51]; however, our study only found an association between GiP initiation and hemoglobin changes from the first, and not from the second, trimester. This could be due to the natural changes in plasma volume and red cell mass that occur during gestation. It is generally accepted that hemoglobin is lowest in the second trimester due to the increase in plasma volume exceeding the increase in red cell mass [52], and then increases from the second to the third trimester due to a rise in erythropoiesis [53, 54]. Our findings suggest that a greater decrease from the first to second trimester is predictive of the initiation of GiP.

Alternatively, it has been speculated that interpersonal differences in hemoglobin, and not simply individual fluctuations, play a greater role in the initiation of GiP [33]. A longitudinal study in Kenya found that individual changes in hemoglobin levels from second trimester were not related to the initiation or cessation of geophagia, but that a one unit increase in average hemoglobin was associated with a 35% decrease in the odds of geophagia [33]. Although our study also found that women with GiP consistently had lower hemoglobin, individual changes might be more significant when they relate to the absence and presence of anemia. Those that initiated geophagia in third trimester experienced an average decline of 1.3 g/dL from 11.7 g/dL in first trimester, almost double what was experienced by those that did not initiate. This would have contributed to many women shifting from above to below the cut-off value for anemia in the third trimester.

The relationship between GiP and iron status could also be more social than physiological. In this cohort, pregnant women were notified and provided supplements if they were diagnosed with low hemoglobin at any ANC visit. Women with anemia may have chosen to eat soil to provide additional mineral supplementation, as has been found among pregnant population groups in South Africa [55], or as a more palatable alternative to the supplements provided by the research team. Additionally, an increase in soil intake in the third trimester, when the fetus is larger in size and pregnancy is more visible, could also be a sign of conformity to cultural beliefs that geophagia is normal and essential for pregnancy [8, 12, 13]. Findings from pregnant women residing in Tanzania’s Geita district reveal that most women practicing geophagia believe it to reduce or stop “morning sickness” [9], however it is worth noting that more than half of these women initiated geophagia in the first, not the third, trimester, when nausea and vomiting is more likely to be experienced [56]. More research is needed on the sociocultural beliefs concerning GiP in Tanzania to inform interventions aimed at preventing this eating behavior from occurring.

The characteristics of GiP have important implications when assessing a pregnant woman’s exposure to trace elements, toxic metals, and soil-borne pathogens. While this study did not involve chemical analysis of the soils ingested, findings from a previous study suggest that soil sticks sold in the Tanga region have concentrations of iron, chromium, and copper that far exceed recommended safety levels [18]. An average intake of 71 g of soil per day, as was found in this study, could result in a high exposure of chromium and copper, which has been shown to restrict fetal growth and increase the risk of pre-term birth [57–59]. Additionally, the preference for hardened soil varieties in this cohort, such as clay and soil-sticks, lends itself to greater tooth abrasion [60]. There is a clear need for clinical controlled trials to clarify the health implications and potential toxicity of GiP.

This study found an association between GiP and iron deficiency before pregnancy. However, since information regarding geophagia before pregnancy was not collected, it cannot be said whether this association is due to a predisposition to low ferritin or because women were used to eating soil prior to conception. Our study also found very few women eating soil in the first or second trimester making it impossible to explore the reverse relationship of GiP on nutritional status. For the same reason, it was not possible to investigate whether changes in nutritional status parameters preceded the initiation of geophagia in the second or first trimester. Future intervention studies should test if earlier access to iron and folate supplementation, for women planning to become pregnant, reduces GiP. It is also worth noting that only 55% of the cohort completed a dietary recall in all three trimesters which limits our sample size for the longitudinal analyses. However the sample size is still greater than what has been used previously to investigate similar research questions [33]. Additional limitations include the lack of vitamin B12, folate, and ferritin measurements in the second trimester and variation in the gestational age where dietary recalls and blood samples were collected. Despite this, efforts were made to include measurements from blood samples that were taken at the time or closest to the time of dietary recall. Strengths of the study include its longitudinal design and the measurement of GiP in early pregnancy, which previous studies have not performed. This is also the first study to have investigated the relationship between various nutritional status parameters and GiP both at pre-pregnancy and throughout the gestational period.

## Conclusions

In conclusion, we found GiP to be associated with lower folate, ferritin and hemoglobin in pregnancy and that a decline in hemoglobin and folate precedes the initiation of geophagia in third trimester. This is the first study investigating GiP in relation to MUAC and vitamin B12 status of which no associations were found. Public health initiatives aimed at preventing GiP should focus on monitoring folate and iron status during pregnancy and ensuring adequate stores prior to conception, either through supplementation, food fortification, or dietary advice. Further research is needed to determine the health consequences of GiP on both mother and offspring and the factors underlying the relationship between GiP, folate and iron status.

## Electronic supplementary material

Below is the link to the electronic supplementary material.

Supplementary Material 1

Supplementary Material 2

## Data Availability

The datasets analysed during the current study are available from the corresponding author on reasonable request. The analytic code will be made publicly and feely available without restriction at https://github.com/LoosTeam/EEberl_GiP.

## Abbreviations

ANC: Antenatal care
FOETALforNCD: FOetal exposure and Epidemiological Transition the role of Anemia in early Life for Non-Communicable Diseases in later life study
GiP: Geophagia in pregnancy
MUAC: Mid-upper arm circumference
SES: Socioeconomic status
