# Supplementary material for: Geophagia in pregnancy and its association with nutritional status - A prospective cohort study in rural north-eastern Tanzania
Source: Int J Behav Nutr Phys Act. 2025 Mar 4;22:27. doi: 10.1186/s12966-025-01721-y (PMC11881378; doi:10.1186/s12966-025-01721-y)
Supplement: Supplementary file 1 — Supplementary Material 1 [file 12966_2025_1721_MOESM1_ESM.docx]

**Supplementary Figure 1.** Pictures of standardized portion sizes of the different soil types consumed. Pictures were used to help specify the type and amount of soil consumed as part of the dietary recall. The weight of the different portion sizes are: Plate -A:150g, -B:100g, -C:60g, -D:25g; Roll -A:36g, -B:25g, -C:17g, -D:9g; Block -A:80g, -B:45g, -C:30g, -D:20g.

**BLOCK A**

**BLOCK A**

**ROLL A**

**PLATE A**

**PLATE B**

**BLOCK B**

**ROLL B**

**BLOCK B**

**PLATE C C**

**BLOCK C**

**ROLL C**

**BLOCK C**

**PLATE D**

**BLOCK D**

**ROLL D**

**BLOCK D**

**Supplementary Figure 2**. Dietary recalls included in cross-sectional analyses and the gestational age that each recall was completed in each trimester.


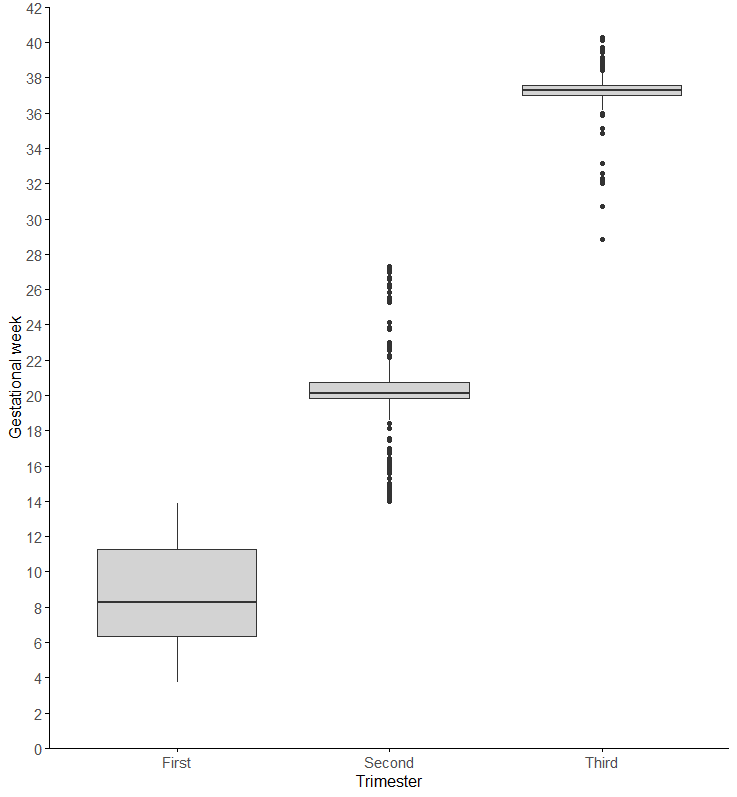
A total of 1237 dietary recalls from 530 women were included in the cross-sectional analyses of geophagia and nutritional status. The gestational age of the women at the time these dietary recalls were completed is shown. First, second and third trimester were defined as <14 gestational weeks, ≥14 and <28 gestational weeks, and ≥28 gestational weeks respectively.

**Supplementary Figure 3.** Days between the dietary recall visits and the nutritional status parameter measurements included in analyses. Measurements of nutritional status parameters: MUAC (purple), vitamin B12 (blue), folate (green), ferritin (yellow), haemoglobin (red), were included in analyses if they were measured in the same ANC visit where women completed a dietary recall, or in the ANC visit preceding or following but within the same trimester (first, second, third).


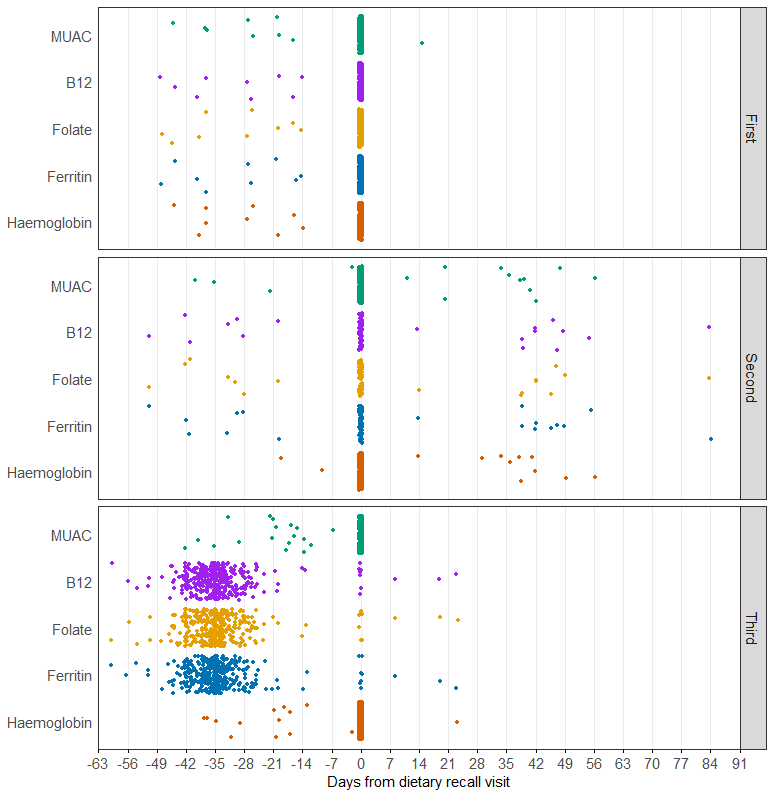


**Supplementary Figure 4.** Trimesters of dietary recall completion in the whole cohort. UpSet plot showing the number of women that completed at least one dietary recall in their first, second, and/or third trimester (n=530).


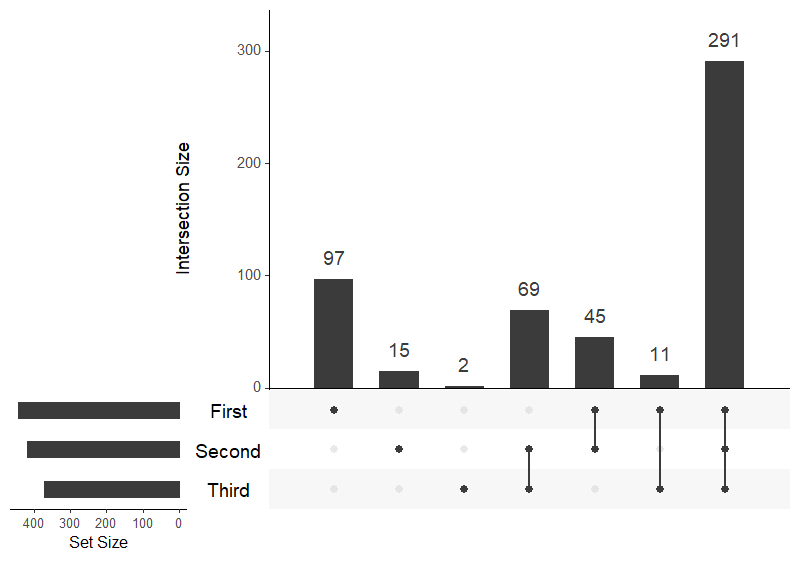


**Supplementary Figure 5.** Trimesters in which geophagia was reported in the whole cohort. UpSet plot showing the number of women that ate soil (geophagia) in the first, second and/or third trimester (n=143). Women completed at least one dietary recall during pregnancy and reported geophagia in at least one of these recalls.


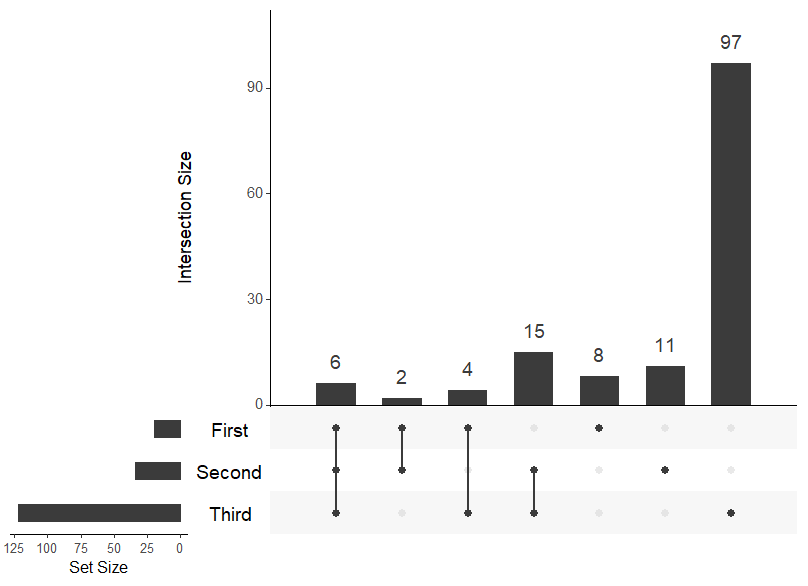


| **Supplementary Table 1.** Association between nutritional status parameters in the first trimester and initiation of geophagia in the third trimester. | | | | | |
| --- | --- | --- | --- | --- | --- |
| **Parameter***^1^* | **Geophagia initiated** | | | | **p-value***^3^* |
|  | **n** | **No***^2^* | **n** | **Yes***^2^* |  |
| **MUAC** (cm) | 188 | 28.2 [27.6; 28.7] | 69 | 28.3 [27.2;29.4] | 0.851 |
| **Vitamin B12** (pmol/L) | 184 | 446.6 [418.6, 474.6] | 70 | 456.5 [405.9, 507.1] | 0.735 |
| Deficient (<150pmol/L) |  | 3 (1.6%) |  | 0 (0%) | 0.671 |
| **Folate** (nmol/L) | 183 | 35.2 [33.5, 37.0] | 68 | 37.3 [33.0, 41.6] | 0.373 |
| Deficient (<10nmol/L) |  | 1 (0.5%) |  | 0 (0%) | 1.000 |
| **Ferritin** (µg/L) | 184 | 39.4 [32.5, 46.2] | 70 | 39.1 [27.8, 50.4] | 0.824*^4^* |
| Deficient (<15µg/L) |  | 51 (28%) |  | 21 (30%) | 0.838 |
| **Haemoglobin** (g/dL) | 188 | 11.8 [11.6, 12.0] | 70 | 11.7 [11.4; 12.0] | 0.910 |
| Anemia (<11g/dL) |  | 41 (22%) |  | 17 (24%) | 0.798 |
| *^1^*Nutritional status parameters measured in the first trimester. Ferritin was corrected for inflammation using a correction factor of 0.67 if CRP > 0.5mg/L. MUAC = mid upper arm circumference. | | | | | |
| *^2^*Mean [95%CI] or frequency (%). | | | | | |
| *^3^*Welch two sample t-test for continuous variables; Pearson's χ2-squared test for categorical variables. | | | | | |
| *^4^*p-value after log-transformation. | | | | | |

| **Supplementary Table 2.** Association between MUAC and haemoglobin in the second trimester and initiation of geophagia (GiP) in the third trimester. | | | |
| --- | --- | --- | --- |
| **Parameter***^1^* | **No GiP***^2^*  (n=188) | **Initiated GiP***^2^*  (n=71) | **p-value***^3^* |
| **MUAC** (cm) | 27.9 [27.4, 28.4] | 28.0 [27.0, 29.0] | 0.939 |
| **Haemoglobin** (g/dL) | 11.1 [10.9, 11.2] | 10.7 [10.4, 10.9] | **0.012** |
| Anemia (<10.5g/dL) | 73 (39%) | 36 (51%) | 0.113 |
| *^1^*Nutritional status parameters measured in the second trimester. MUAC = mid upper arm circumference. | | | |
| *^2^*Mean [95%CI] or frequency (%). | | | |
| *^3^*Welch two sample t-test for continuous variables; Pearson's χ2-squared test for categorical variables. P-values <0.05 are shown in bold. | | | |

**Supplementary Table 3.** Changes in MUAC and haemoglobin from first to second trimester and its association with the initiation of geophagia (GiP) in the third trimester.

| **Parameter***^1^* | **No GiP***^2^*  (n=188) | **Initiated GiP***^2^* | **OR**  **[95%CI]** | **AOR [95%CI]***^3^* | **AOR**  **p-value** | |
| --- | --- | --- | --- | --- | --- | --- |
| **MUAC**  (cm) | -0.24  [-0.41;-0.08] | -0.36  [-0.60;-0.11] | 0.91 [0.72;1.17] | 0.90 [0.70;1.16] | | 0.406 |
| **Haemoglobin** (g/dL) | -0.71  [-0.86;-0.55] | -1.08  [-1.30;-0.85] | 0.68 [0.50;0.92] | 0.64 [0.46;0.88] | | **0.006** |
| *^1^*Nutritional status parameters measured in the second and third trimesters. MUAC = mid upper arm circumference. | | | | | | |
| *^2^*Mean difference [95%CI] from first to second trimester. Sample size for women who initiated GiP: MUAC n=69, haemoglobin n=70. | | | | | | |
| *^3^*AOR= Adjusted for civil status, gestational age (days) at enrollment, and Hemovit or vitamin B12 supplementation during pregnancy. | | | | | | |
